# A 5-day cytoreductive chemotherapy followed by haplo-identical hsct (FA5-BUCY) as a tumor-ablative regimen improved the survival of patients with advanced hematological malignancies

**DOI:** 10.18632/oncotarget.12383

**Published:** 2016-10-01

**Authors:** Ting Yang, Qiaoxian Lin, Jinhua Ren, Ping Chen, Xiaohong Yuan, Xiaofeng Luo, Tingbo Liu, Jing Zheng, Zhihong Zheng, Xiaoyun Zheng, Xinji Chen, Langhui Zhang, Hao Zheng, Zaisheng Chen, Xueling Hua, Shaohua Le, Jian Li, Zhizhe Chen, Jianda Hu

**Affiliations:** ^1^ Department of Hematology, Fujian Institute of Hematology, Fujian Provincial Key Laboratory on Hematology, Fujian Medical University Union Hospital, Fuzhou 350001, Fujian, P. R. China

**Keywords:** haplo-identical HSCT, cytoreductive chemotherapy, refractory, hematological malignancies, disease control

## Abstract

Haplo-HSCT has been used when HLA-matched siblings are not available. Conditioning regimens aim to reduce tumor burden prior to HSCT and provide sufficient immunoablation. We report the outcome of haplo-HSCT in 63 consecutive patients from 2/2013 to 12/2015 (19 females/44 males) with high-risk or relapsed/refractory hematological malignancies (n=29-AML; 8-sAML; 19-ALL; 5-advanced-MDS; 2-CML-BC). Median age was 20 years (range: 1.1-49). Twenty-one patients achieved remission prior to transplant, while 42 did not. Patients received FA5-BUCY, i.e., 5-day salvage chemotherapy (Fludarabine/Ara-C) and conditioning (Busulfan/Cyclophosphamide). GvHD prophylaxis included ATG, CsA, MMF and short-term MTX. All patients received stem cells from bone marrow and peripheral blood, and achieved successful engraftment, except two who died before. With a median follow-up of 269 days (120-1081), 42/63 patients are still alive and disease-free. Two-year OS and RFS were similar in patients not in remission and in those in complete remission (61.3% vs 56.3%, *p*=0.88; 58.3% vs 56.3%, *p*=0.991). Non-relapse mortality and relapse incidence were 22.2% and 11.1%, respectively. Severe acute-GvHD occurred in 4/63 patients. Transplant-related mortality was low at day+100 (17.5%) and for the entire study period (20.6%). Unexpectedly, few patients experienced mild-to-moderate toxicity, and main causes of death were infection and GvHD. BM blast counts, age, and donor-recipient gender-pairs did not affect the outcome. Less chemotherapy cycles prior to HSCT might result in more favorable outcome. Thus, haplo-HSCT with FA5-BUCY appears promising for advanced disease, especially when TBI and amsacrine, used for FLAMSA, are not available and in pediatric patients for whom TBI is not recommended.

## INTRODUCTION

Despite significant advances in the treatment of leukemia, prognosis of patients with refractory or relapsed disease remains poor. Allogeneic hematopoietic stem cell transplantation (allo-HSCT) is now considered as the treatment with the highest probability of cure for primary refractory or relapsed leukemia [1]. As the availability of an HLA-matched sibling constitutes a barrier, especially in China because of the 1-child policy of the past 30 years, studies have shown that haploidentical HSCT (haplo-HSCT) with a graft from a partially matched family member is a possible alternative. In fact, a recent study in China by Huang *et al.* demonstrated that outcomes similar to those of identical-sibling transplant could be achieved with haplo-HSCT in patients with acute myeloid leukemia (AML) in first remission [2].

Preparative conditioning regimens prior to cell infusion are critical for reduction of tumor burden and immunoablation [3]. While earlier conditioning regimens used high-doses total body irradiation (TBI) and chemotherapeutic agents (myeloablative), new reduced-intensity conditioning regimens have been proposed after the recognition that graft-versus-tumor effects contributed to the effectiveness of transplantation. Such protocol, i.e., the FLAMSA regimen, initially proposed by Schmid and colleagues [4] and consisting of sequential administration of aplasia-inducing chemotherapy (fludarabine, Ara-C, and amsacrine) followed by TBI plus cyclophosphamide (TBI/CY) or busulfan/CY (BU/CY), was found to be very effective for high-risk, refractory or relapsed AML [4–9]. However, this regimen could not be used at our center as amsacrine was not available in China, and we are not equipped to administer TBI.

As highly effective or standardized conditioning regimens have not been extensively tested for high-risk, relapsed/refractory leukemic patients, we applied the similar sequential treatment strategy as FLAMSA using a 5-day course of fludarabine and Ara-C for cytoreduction in the first phase, followed by BU/CY (named FA5-BUCY), and investigated the efficacy and safety of this regimen in patients with high-risk and advanced hematological malignancies. Here, we report the outcome of this cohort of 63 patients who were treated with haplo-HSCT after FA5-BUCY. The data show that FA5-BUCY is safe and can be successfully applied to relapsed/refractory patients who did not reach remission before transplantation.

## RESULTS

Remission was achieved in 21 patients prior to the transplant, while 42 patients had no evidence of remission. Leukemic blasts in the bone marrow ranged from 7%-98% (median: 35%) in the patients who did not achieve hematological remission.

### Engraftment and donor chimerism

All patients received fresh grafts containing a median of 7.9×10^8^ mononuclear cells/kg (range 4.2-21.4×10^8^/kg) and 5.33×10^6^ CD34+ cells/kg (range 2.6–28×10^6^/kg) in total from BM and PB on day 0. The time to neutrophil and platelet engraftment was 13 days (range 10–25) and 13 days (range 7–40), respectively. Two patients were not evaluable as one died before engraftment due to cerebral hemorrhage and the other died of severe bloodstream infection with both pan-resistant *Pseudomonas Aeruginosa* and *Candida Tropicalis*. For the other 61 patients, 95.1% had complete donor chimerism at day 30 and thereafter, and 4.9% had mixed donor/recipient chimerism, which eventually converted to full donor chimerism during the 6 months follow-up.

### Toxicity

Eighteen patients (28.6%) experienced cytarabine syndrome (fever, maculopapular rash and conjunctivitis), which was easily controlled with corticosteroids. Gastrointestinal (GI) disturbances, which commonly include symptoms of anorexia, vomiting and diarrhea, occurred in some cases, but were very mild and did not generally need using total parenteral nutrition (TPN) support. Ten patients (15.9%) developed toxic liver dysfunction, 7 with grade I hepatic toxicity, and 3 with grade II. Increase in bilirubin was observed, with a median value of 3.4 mg/dl (range, 2.1-6.2 mg/dl) and a median peak occurring on day 9 (range, days 6-15). All the patients who had hepatitis recovered fully and promptly with routine supportive treatment. None of the patients had severe organ failure.

### Overall outcome

At the time of engraftment, 60 of the 61 patients (98.4%) achieved complete remission, while one (1.6%) had still not achieved remission even through engraftment was successful. At the time of analysis, 42/63 patients (66.7%) were still alive and in complete remission (CR), with a median follow-up time of 269 days (range:120-1081). Among these 42 patients, 30 patients (71.4%) had not achieved remission before transplant. Seven patients (11.1%) relapsed at median 133 days after transplant (range: 63-312).

Twenty-one patients died. The causes of death are summarized in Table 1. Fourteen patients (22.2%) died from NRM, mainly from GvHD or infections. One patient (1.6%) had secondary graft failure (GF) with uncontrolled cytomegalovirus (CMV) infection. Three patients died from cerebral hemorrhage (n=1), suicide (n=1) and electrolyte imbalance (n=1). The 100-day mortality and TRM were 17.5% and 20.6% (excluding one case of suicide), respectively.

**Table 1:** Causes of death in the 63 consecutive patients

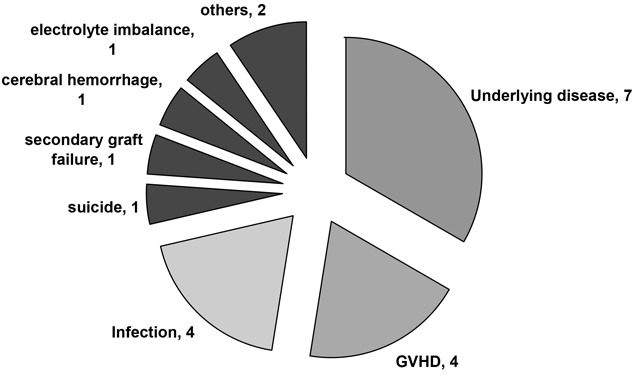

Twenty-one patients (21/63) died. Seven patients (7/21) died of relapse. Fourteen patients (14/21) died from NRM, mainly from GvHD or infections. One patient had secondary graft failure (GF) with uncontrolled cytomegalovirus (CMV) infection. Three patients died from cerebral hemorrhage (n=1), suicide (n=1) and electrolyte imbalance (n=1).

### GvHD

The incidence of acute (a) and chronic (c) GvHD is summarized (shown in Supplementary Table S1). Eight patients (8/63) had grade I-II aGvHD. One patient had stage I skin GvHD, one had stage II gut GvHD, five had stage II aGvHD involving skin and liver, and one involving skin, liver and GI tract. Four patients (4/63) had grade III-IV aGvHD, either involving liver or GI tract. Four of these 12 patients died of complications related to severe aGvHD, and 1 died of relapse despite the presence of moderate aGvHD. All others who developed aGvHD are currently alive and disease-free. Six patients (9.5%) had limited cGvHD. Among the patients with GVHD, 2 out of 10 patients who received pDLI had grade I-II aGvHD, one had limited cGvHD and one patient died of complications related to grade IV aGvHD involving the GI tract.

The major therapy administered for GvHD was systemic corticosteroids, whereas monoclonal antibodies (Basiliximab, Etanercept, and others) were used in 4 steroid-refractory cases.

### Disease control based on BM cellularity and minimal residual disease

MRD assessment via WT1 expression and flow cytometry has been shown to correlate with disease status, and along with marrow blast counts, is a rapid and sensitive measure of treatment efficacy. Ten out of 42 patients who were not in remission were evaluated for disease response at different time points during HSCT (T I-IV; see Methods). They all exhibited disease prior to transplantation (T I) with median marrow cellularity of 50% (range 10–90%), overexpression of WT1 and positive MRD. However, after FA5-BUCY conditioning (T III; Table 2), cytoreduction was successfully achieved in all 10 patients who demonstrated extreme hypocellularity of the bone marrow (range 0–30%), reduced expression of WT1 below the cutoff value (50%) [10, 11, 12], and negative MRD (<10^−4^) [13, 14], followed by a further decrease or stabilization after the engraftment (T IV). Only 1 patient (patient 4) showed a significant transient increase in BM blasts and WT1 expression, and a positive MRD after the 5-days of intensive chemotherapy with FA; however, a further decrease was observed after administration of BUCY.

**Table 2:** BM cellularity and MRD assessment at different times during HSCT

No. of Cases	Diagnosis	Blast (%)				MRD/Flow				WT1 (%)			
		T I	T II	T III	T IV	T I	T II	T III	T IV	T I	T II	T III	T IV
1	AML	36.5	14	7	0.5	4.4×10^−1^	1.05×10^−1^	<10^−4^	<10^−4^	88.51	56.38	12.88	38.02
2	AML	12.5	—	—	1	1.24×10^−2^	<10^−4^	<10^−4^	<10^−4^	93.11	50.77	2.5	0.37
3	AML	8	—	—	0	4×10^−2^	5×10^−3^	<10^−4^	<10^−4^	57.39	16.50	8.84	0.08
4	ALL	23.5	79	0.5	2	9×10^−2^	3×10^−1^	<10^−4^	<10^−4^	79.39	71.66	12.47	0.08
5	ALL	39.5	23	—	1.5	2.8×10^−1^	6×10^−2^	<10^−4^	<10^−4^	97.01	50.3	23.6	0.07
6	sAML(MDS)	98	40.5	15.5	2.5	1×10^−1^	8×10^−3^	<10^−4^	<10^−4^	95.06	84.05	27.37	3.41
7	AML	95	40	18	2	8.2×10^−1^	4.88×10^−1^	3.5×10^−2^	<10^−4^	95.68	83.5	72.01	42.06
8	sAML(MDS)	11	8.5	—	1.5	3.9×10^−1^	4.3×10^−1^	<10^−4^	<10^−4^	65.2	46.44	30.9	11.70
9	AML	45	—	—	0.5	4.43×10^−1^	<10^−4^	<10^−4^	<10^−4^	84.32	21	11.5	0.52
10	AML	88	10	—	0.5	54.91×10^−1^	3.9×10^−1^	<10^−4^	<10^−4^	74.44	30.9	11.7	0.15

Ten evaluable patients exhibited disease at the time of transplantation as shown by WT1 overexpression and positive MRD. The disease responses were measured at different time points defined as: time point I (T I), before the initiation of administration of fludarabine and cytarabine; time point II (T II), right after completion of the 5-days FA; time point III (T III), right after conditioning with BUCY; and time point IV (T IV), at the time of hematological engraftment. A decrease in the number of marrow blasts (<5%), downexpression of WT1 below the cutoff value (50%), and negative MRD (<10^−4^) were documented after the FA5-BUCY conditioning, followed by a further decrease or stabilization after the engraftment. Only 1 patient (patient 4) showed a significant transient increase of marrow blasts, WT1 overexpression and positive MRD after the 5-days intensive chemotherapy of FA, then a further decrease after the BUCY regimen.

### Overall survival (OS) and relapse-free survival (RFS)

**Figure 1:**
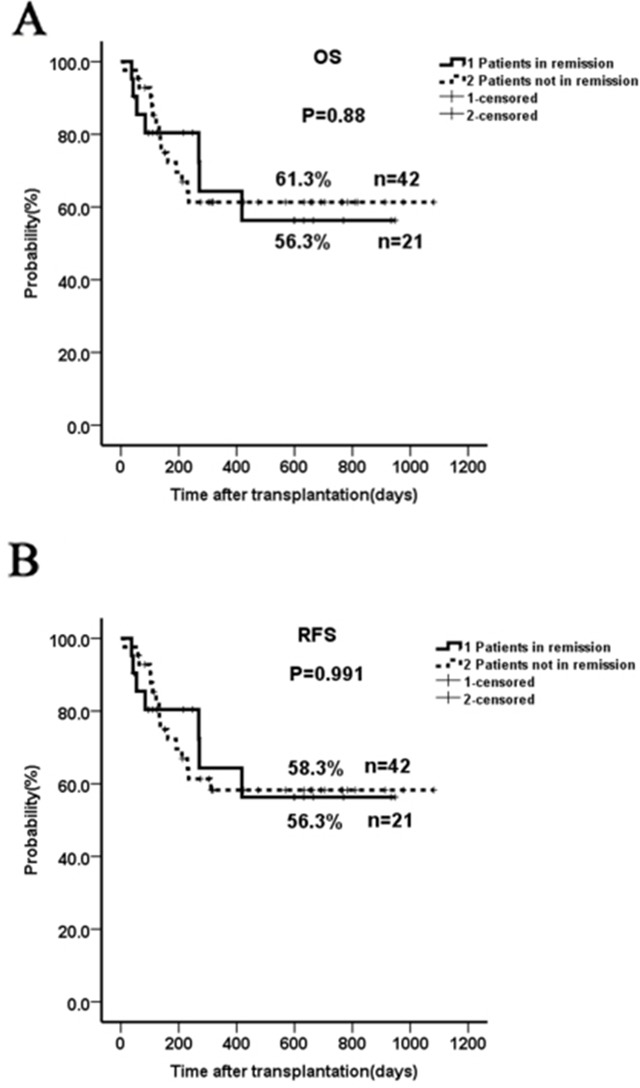
Two-year OS and RFS in patients not in remission prior to HSCT compared to those in complete remission The two-year OS and RFS in patients not in remission were similar to those from patients in complete remission (61.3% vs 56.3%, *p*=0.88; 58.3% vs 56.3%, *p*=0.991).

**Figure 2:**
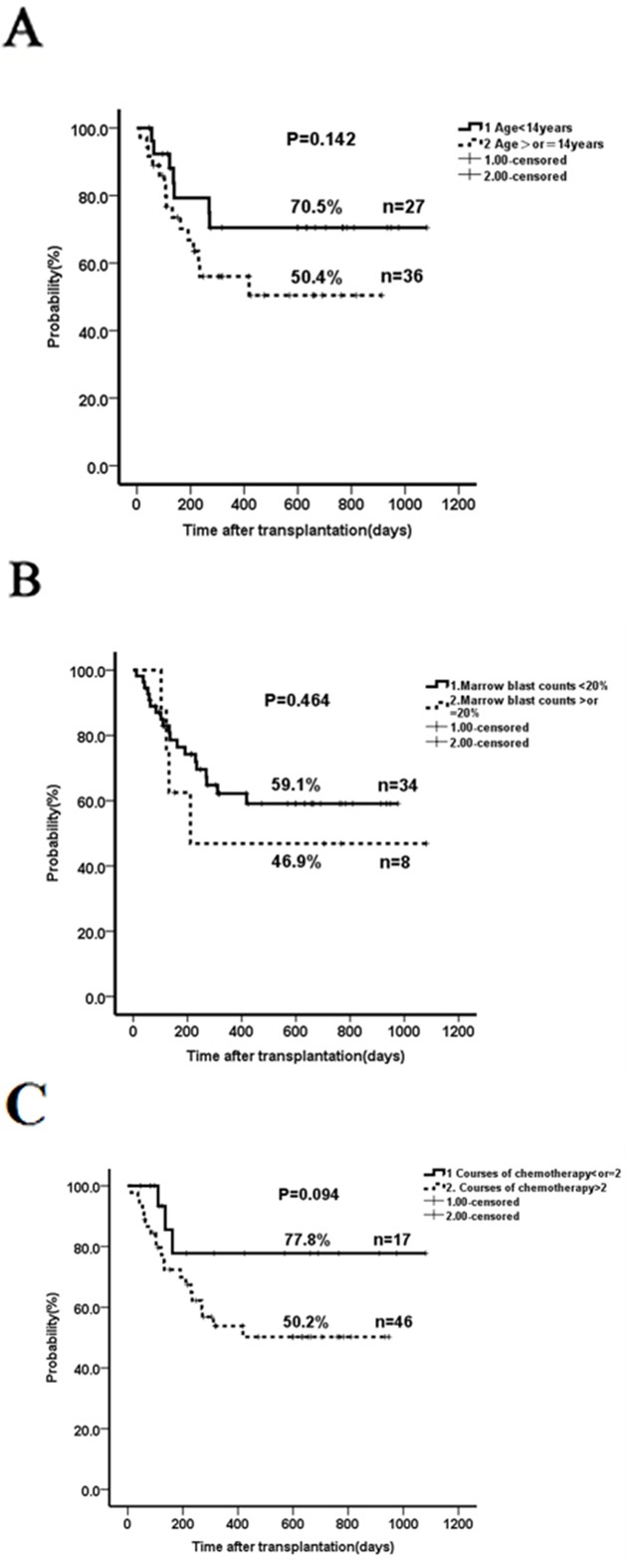

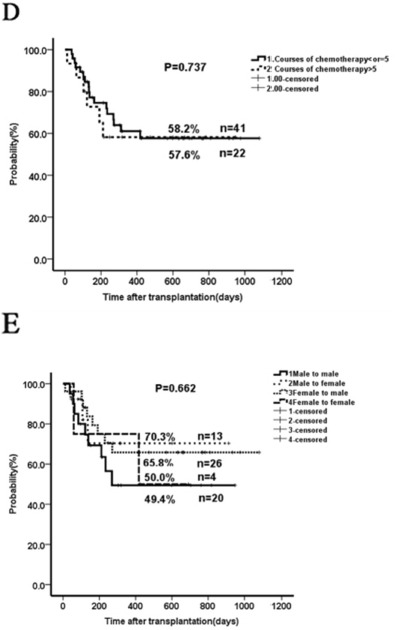
The impact of age, marrow blasts, previous cycles of chemotherapy and donor-recipient gender pairs on overall survival **A.** Two-year OS in patients with age ≥14 years compared to those with <14 years. **B.** Two-year OS in patients with marrow blast count ≥20% compared to those with <20%. **C.** Two-year OS in patients who received ≤2 cycles of chemotherapy prior-HSCT compared to those with >2 cycles. **D.** Two-year OS in patients who received > 5 cycles of chemotherapy prior-HSCT compared to those with ≤ 5 cycles. **E.** Two-year OS in patients who received different donor-recipient gender pairs. The outcome in the subgroup ≥14 years (n=36) was not significantly different than that in patients younger than 14 years of age (n=27) when taking into account all 63 patients (2-year OS of 50.4% versus 70.5 %, p=0.142; Figure 2A). Patients with a marrow blast count ≥20% (n=8) prior to conditioning had a comparable outcome as those with marrow blasts <20% (n=34) (2-year OS 46.9% vs 59.1%, p=0.464; Figure 2B). Patients who received ≤2 cycles (n=17) of chemotherapy before haplo-HSCT seemed to have a better OS than those with >2 cycles (n=46) (2-year OS 77.8% vs 50.2%, p=0.094; Figure 2C); however, no significant difference was seen when comparing > 5 cycles (n=22) and ≤ 5 cycles (n=41) (Figure 2D). There was no significant impact of donor-recipient gender pairs on OS (Figure 2E).

The two-year OS and RFS in patients not in remission were similar when compared to those in complete remission (61.3% vs 56.3%, *p*=0.88; 58.3% vs 56.3%, *p*=0.991, respectively; Figure 1). The outcome in the subgroup ≥14-years (n=36) was not significantly different than that in patients younger than 14 years of age (n=27) when taking into account all 63 patients (2-year OS of 50.4% versus 70.5 %, *p*=0.142; Figure 2A) or only the subgroup not in remission (data not shown). Patients with a marrow blast count ≥20% (n=8) prior to conditioning had a comparable outcome as those with marrow blasts <20% (n=34) (2-year OS 46.9% vs 59.1%, *p*=0.464; Figure 2B). Patients who received ≤2 cycles (n=17) of chemotherapy before haplo-HSCT seemed to have a better OS than those with >2 cycles (n=46) (2-year OS 77.8% vs 50.2%, *p*=0.094; Figure 2C); however, no significant difference was seen when comparing ≥ 5 cycles (n=22) and < 5 cycles (n=41) (Figure 2D). Contrary to what was previously reported [15], there was no significant impact of donor-recipient gender pairs on OS in our study (Figure 2E). Although the numbers were too small for statistical studies, GvHD did not seem to affect outcome (data not shown). Thirty-two out of the 42 patients not in remission did not receive DLI post-HSCT due to the presence of active infection, aGvHD and other disorders; however, there was no significant difference in the 2-yr RFS when compared to patients who receive DLI (Supplementary Figure S1).

### Comparison with historical control

Conditioning regimens such as BUCY or CY-TBI are considered as the conventional conditioning regimens for allo-HSCT in patients with leukemia or MDS. Tang *et al.* compared their study of allo-HSCT in AML patients to a historical control series composed of 26 patients who received allo-HSCT between January 2000 and May 2011 after conventional conditioning regimens [9]. To demonstrate the effectiveness of FA5-BUCY in the 42 patients not in remission from our study group, we similarly compared the outcome of our patients to that of this previously reported historical control series [9]. Patient characteristics, GvHD prophylaxis and graft sources were comparable in both groups (Table 3). Although the tumor burden evaluated by marrow blasts was much higher in our study group, preparation with the FA5-BUCY regimen resulted in significantly improved 2-year relapse rates (11.9% vs 81.2%, p<0.001), NRM (26.2% vs 40.9%, p<0.001), RFS (58.3% vs 11.11%, p<0.001) and OS (61.3% vs 11.11%, p<0.001).

**Table 3:** Characteristics and transplant outcome of patients not in remission in our study group and historical group^9^

	Study group (42 cases)	Historical group (26 cases)	P value
Age, median (range)	23 (1.1-49)	35 (18-5)	
Sex			
Male	31	15	0.197
Female	11	11	
Marrow blasts (%)			0.002
Blasts < 5%	0	6	
Blasts ≥ 5%	42	20	
Graft sources			
BM	0	1	
PBSC	0	25	
BM+PBSC	42		
GvHD prophylaxis			
ATG+CSA+MMF+MTX	42 (100%)	26 (100%)	
Transplant outcome (2-years)			< 0.001
Relapse rate	11.9%	81.2%	
Non relapse mortality	26.2%	40.9%	
Relapse free survival	58.3%	11.11%	
Overall survival	61.3%	11.11%	

## DISCUSSION

Complete remission is rarely achieved in patients with relapsed or refractory acute leukemia despite the development of new investigational therapies. Allo-HSCT is currently the treatment with the highest probability of cure, allowing the effective elimination of the leukemia-initiating cells. However, the disease status prior to HSCT, often uncontrolled, has limited the overall survival, even among younger patients with a well-matched donor [16–18]. With conventional conditioning regimens such as BUCY or CY-TBI, most patients who were not in remission at the time of HSCT relapsed rapidly within 6 months of transplantation, as previously reported [9] and as we have experienced (personal communication). Nevertheless, sequential treatment strategies including intensive chemotherapy to reduce disease burden followed by reduced-intensity conditioning have been prospectively shown to improve treatment outcome in leukemias, especially for high-risk, relapsed/refractory patients with active disease [4–9, 19]. Among those, the FLASMA-based protocol (fludarabine/amsacrine/cytarabine) has been extensively used with great success. Furthermore, a novel active agent, clofarabine, which has no overlapping toxicities to the conditioning regimen, has been used as a pre-conditioning, intensification cytoreduction agent [19]. Here, we evaluate the efficacy and safety profile of the FA5-BUCY regimen in 63 acute leukemic patients either at high risk (n=21) or not in remission (median marrow blasts of 35% pre-HSCT, n=42). All these patients could not receive HLA identical allo-grafts due to the lack of HLA identical siblings.

The combination of fludarabine and Ara-C/cytarabine in a FLAG-based regimen has been used as the salvage treatment for patients with relapsed/refractory leukemia and is well-tolerated, although as a second-line chemotherapy regimen, it has limited efficacy on achieving remission for relapsed/refractory patients [20, 21]. Our analysis demonstrated that the Regimen of a 5-day course of FA combined with Bucy still resulted in significant cytoreduction prior to HSCT, as evidenced by a decrease in BM blasts and MRD, It was previously reported that for each 10% increase in marrow blasts at the time of HSCT, there was an increased risk of death by a factor of 1.21 [22]. Thus, the early removal of blasts by the combination of FA with Bucy before stem cell infusion may offer benefits, resulting in transplant tolerance and better survival [23, 24] Our study enrolled 42 patients with molecular or hematological (7-98% BM blasts) advance disease prior to HSCT, who had relapsed or were refractory to the standard frontline treatment, and did not respond to multiple cycles of salvage treatments. These patients are usually not considered as good candidates for transplantation, however, our results revealed that HSCT with FA5-BUCY conditioning brought about a rapid MRD remission and efficiently reduced tumor burden before stem cell infusion. Moreover, 98.4% did achieve complete remission at the time of engraftment, and the reduction of disease burden had a favorable impact on outcome. The 2-year OS (61.3%) and RFS (58.3%) were similar in patients not in remission before HSCT compared to patients in remission. There may be a statistical bias because of the lower number of patients in the remission group, nevertheless, the OS were better than those reported in previous studies, which is very encouraging for treating these patients [4–9, 19]. Although haploidentical transplantation might be expected to be associated with lower relapse rate and disease-free survival because of a more potent graft-versus-leukemia (GVL) effect, and no difference in relapse rate was found whether or not DLI was used, we believe that decreasing tumor burden with FA5-BUCY is the key factor to improve outcome, especially when compared to the outcomes following conventional conditioning regimens. Importantly, these favorable outcomes were not affected by the blast counts in the bone marrow, the age of the patients or the presence of GvHD, indicating that haplo-HSCT with FA5-BUCY regimen is beneficial to the high-risk, relapsed/refractory leukemic patients regardless of the percentage of bone marrow blasts at the time of HSCT. In addition, it is believed that children have a much better prognosis than adults because children usually do not have the comorbidities typically found in adults (e.g., chronic hypertension, diabetes mellitus, chronic coronary ischemic disease, smoking-related illnesses). Our results indicate that FA5-BUCY regimen can be used in pediatric or adult patients, especially in pediatric patients for whom TBI is not recommended.

As chemotherapy can also affect the rapidly growing and dividing normal cells, such as those in the bone marrow, digestive tract, skin, hair and reproductive organs, less chemotherapy may be helpful to minimize the risk of infection, graft failure and GvHD, and such protocols merit further investigation. Our data reveal that less cycles of chemotherapy prior to HSCT may lead to a more favorable clinical outcome. Thus, it suggests that transplant decision should be made as early as possible once the patients start developing resistance to the chemotherapy. For patients who do not have a matched sibling donor, our data show that a haploidentical family donor can be an alternative option. As a parent is always a haploidentical match for the children and vice versa, it is reasonable to test the close family members early to proceed quickly with this life-saving transplant and avoid the waiting periods often occurring with unrelated donors. Interestingly, our analysis did not find any negative impact of patient-donor gender pairs on transplant outcome (Figure 2E), contrary to previous reports [15, 25, 26]. It indicates that the donor source is not a major limitation, making haplo-HSCT a more feasible alternative.

While FA5-BUCY is an intensive regimen, it was unexpectedly associated with only mild to moderate toxicity. According to the assessed EBMT risk score, most of the patients enrolled in the study were fit and suitable for allo-HSCT as recommended [1]. One of the most important findings in the present analysis is that the probability of TRM in such a high-risk cohort was low, both at day +100 (17.5%) and for the entire study period (20.6%), and that the majority of deaths was attributed to early transplant-related complications, which may be associated with active disease. The major side effects of FA5-BUCY were gastrointestinal and hepatic toxicity, which can be resolved within a relatively short time after prompt and appropriate management. The fact that only rare cases of severe mucositis, a common complication post-HSCT related to high-intensity conditioning [9, 27, 28] were observed, resulted in better quality of life, treatment adherence, and ultimately improved outcome in these patients.

Taken together, our data demonstrate that FA5-BUCY as an intensive conditioning regimen prior to haplo-HSCT presents advantages for leukemic patients who did not reach remission, and does not increase NRM. While we didn't compare haplo to matched donors in our current study, we suggest that haplo-SCT can be an alternative option, including with the mother as the donor, especially when the refractory patients urgently need to undergo this salvage therapy. In addition, haplo-SCT can be performed rapidly because the donors are all close family members. As the heterogeneity of the data and the size of the population may have been limiting factors, further investigations with larger cohorts are warranted.

## MATERIALS AND METHODS

### Study design

We conducted a nonrandomized, observational study from a single center in a cohort of high-risk, primary refractory or relapsed patients with hematological malignancies who underwent conditioning with the FA5-BUCY regimen, registered on http://ClinicalTrials.gov (NCT02328950), and haplo-HSCT at the Union Hospital, Fujian Medical University, Fujian Province, China between Feb. 2013 and Dec. 2015. Informed consent was obtained from all patients and donors before being included in the study. This study was conducted in accordance with the ethical standards of the local institutional review board and with the Helsinki Declaration.

### Patient characteristics

Patient characteristics are summarized in Table 4. Sixty-three consecutive patients (19 females, 44 males) with a median age of 20 years (range 1.1–49) were included in the study. 42 patients had relapsed (n=34) or primary refractory (n=8) leukemia, and 21 patients had leukemia in remission but had a high risk of relapse per cytogeneics and/or molecular markers based on the National Comprehensive Cancer Network practice guidelines [29]. All patients had received standard frontline treatment. After failure of more than 2 cycles of salvage treatments, 15/42 patients had relapsed/refractory diseases without molecular remission, and 27/42 had hematological disease with 7%-98% BM blasts (median 35%) prior to HSCT”.

**Table 4:** Patient Characteristics

Median age, years (range)	20 (1.1-49)
Sex	
Male	44 (69.8%)
Female	19 (30.2%)
Primary disease	
AML	29 (46.0%)
MDS-SAML	8 (12.7%)
ALL	19 (30.2%)
Advanced MDS	5 (7.9%)
CML-BC	2 (3.2%)
Remission status	
In remission	21 (33.3%)
Not in remission	42 (66.7%)
Donor-recipient relationship	
Parents-Children	36 (57.1%)
Siblings	21 (33.3%)
Children-Parents	6 (9.5%)
Donor-recipient blood type	
Compatibility	34 (54.0%)
Major ABO incompatibility	11 (17.5%)
Minor ABO incompatibility	15 (23.8%)
Major and minor ABO incompatibility	3 (4.8%)
EBMT risk scores	
0-2	36 (57.1%)
3-4	23 (36.5%)
5-7	4 (6.3%)
Donor-recipient gender pairs	
M to M	20 (31.7%)
M to F	13 (20.6%)
F to M	26 (41.3%)
F to F	4 (6.3%)
Engraftment	
Neutrophils, day (range)	13 (10-25)
Thrombocytes, day (range)	13 (7-40)
Infusion dose CD34+, 10^6^/kg (range)	5.33 (2.6-28)
Follow-up time, days (range)	269 (120-1081)

Primary refractory AML was defined as blast persistence in bone marrow (BM) aspirates on day 28 after the first or second induction treatment. Relapsed AML was defined as >5 % blasts in BM aspirates in patients who achieved complete cytological remission after the first or second induction treatment. The diagnoses were as follows: acute myeloid leukemia (AML, n=29), AML secondary to myelodysplastic syndrome (sAML, n=8), acute lymphoblastic leukemia (ALL, n=19), advanced myelodysplastic syndrome (aMDS, n=5) and chronic myelogenous leukemia in blast crisis (CML-BC, n=2).

### HLA typing and donors

Patients and their donors were tested for HLA-A, HLA-B, HLA-C, HLA-DRB1, and HLA-DQB1 by high-resolution molecular typing methods. All donors were family members who shared one HLA haplotype with the recipient, but differed to various degrees for the HLA-A, B, and DR antigens of the unshared HLA haplotype. Parents accounted for 57.1% of the donor sources.

All patients received both BM and peripheral blood (PB) mobilized with granulocyte colony-stimulating factor (G-CSF; 5 μg/kg of body weight per day for 5 days), as described by Huang *et al* [2]. The target mononuclear cell counts in total from BM and PB were >4 × 10^8^/kg of recipient weight.

### EBMT risk score

To assess the risks of haplo-HSCT, the European Group for Blood and Marrow Transplantation (EBMT) risk score [30] was calculated for each individual patient before transplantation based on 5 pre-transplantation variables: age, disease stage, time from diagnosis to transplantation, donor type, and donor–recipient sex combination.

### Conditioning regimens, transplantation and GvHD prophylaxis

The FA5-BUCY protocol is shown in Table 5. All 63 patients received the aplasia-inducing salvage therapy consisting of 30 mg/m^2^/day Fludarabine and high-dose 2 g/m^2^/day Ara-C (Cytarabine) for 5 consecutive days from day −13 to day −9, followed after 1 day of rest by 3.2mg/kg/day BU from day −7 to day −5 and 1.8g/m^2^/day CY from day −4 to day −3.

**Table 5:** Protocol for FA5-BUCY conditioning regimen

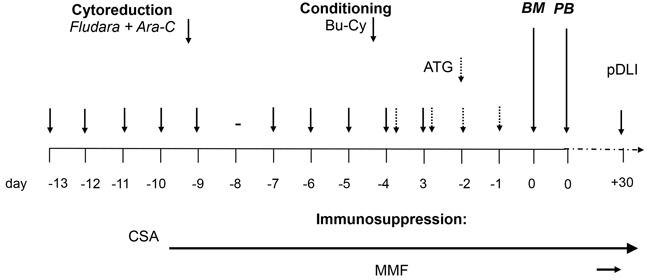

The FA5-BUCY regimen consists of 30 mg/m^2^/day Fludarabine and high-dose 2 g/m^2^/day Ara-C (Cytarabine) for 5 consecutive days from day −13 to day −9, followed after 1 day of rest by 3.2mg/kg/day BU from day −7 to day −5 and 1.8g/m2/day CY from day −4 to day −3. All patients received stem cells from both bone marrow (BM) and peripheral blood (PB). GvHD prophylaxis included ATG, CsA, MMF and short-term MTX.

Prophylactic donor lymphocyte infusions (DLI) were given from day +30 in patients without evidence of graft versus host disease (GvHD) or severe infection, as recommended by Kolb *et al* [31]. Peripheral blood mononuclear cells from donors were cryopreserved and adjusted to a defined CD3+ T lymphocyte count (5×10^5^ to 1×10^6^ CD3 + cells/kg) before transplantation.

GvHD prophylaxis consisted of rabbit anti-thymocyte globulin (20 patients with Thymoglobulin, Genzyme, 10mg /kg and 23 with ATG-Fresenius®, 40 mg/kg) from day -4 to day-1, cyclosporine A (plasma level 100–250 ng/ml, starting from day −10, and tapered from the second or third month if no signs of GvHD were present), mycophenolate mofetil (5 mg/kg bid, starting from day +7 and tapered after engraftment), and short-term methotrexate (MTX, 15 mg/m^2^ at day +1, and 10 mg/m^2^ at day +3 and +6).

Acute GvHD was graded according to the Glucksberg criteria [32]. Chronic GvHD was graded according to the revised Seattle classification [33].

All patients were treated according to our institutional transplant guidelines for antiviral, antifungal, and antimicrobial prophylaxis.

### Toxicity

Regimen-related toxicity was graded according to the scale of the NCI Common Terminology Criteria for Adverse Events (CTCAE) version 4.0 [34, 35].

### Engraftment

Engraftment was assessed by peripheral blood counts. Neutrophil engraftment was defined as the first of two consecutive days with an absolute neutrophil count over 500 neutrophils/L. Platelet engraftment was defined as the first of two consecutive days with more than 20,000 platelets/L without platelet transfusion.

The hematopoietic donor cell chimerism status of mononuclear cells and CD3+ T cells was monitored in all patients using microsatellite markers, as described [36].

### Minimal residual disease

To evaluate the cytoreduction effects and disease response of FA5-BUCY regimen, a BM aspirate and a biopsy were performed to evaluate the kinetics of the BM blasts from 10 evaluable patients at different time points defined as: time point I (T I), before the initiation of administration of fludarabine and cytarabine; time point II (T II), right after completion of the 5-days FA; time point III (T III), right after conditioning with BUCY; and time point IV (T IV), at the time of hematological engraftment. At each time point, the Wilms tumor gene 1 (WT1) dynamic expression was also assessed using quantitative RT-PCR [10, 11], and minimal residual disease (MRD) was detected by multiparametric flow cytometry (FC) with a Cytomics FC-500 flow cytometer (Beckman-Coulter, Brea, CA) and the following combinations of monoclonal antibodies (mAbs): FITC, PE, PE-Texas Red, and PE-Cy5: CD13/CD33/CD45/CD34, CD38/CD56/CD45/CD34, CD15/CD14/CD45/CD34, CD15/CD13/CD34/CD33, CD64/CD11B/CD45/CD34, HLA-DR/CD117/CD34/CD33, CD5/CD117/CD34/CD33, CD7/CD34/CD45/CD2, CD20/CD19/CD34/CD45, CD41/CD235a/CD45/CD34, and CD45/CD4/CD8/CD3. A percentage of leukemic cells exceeding 10^−4^ was considered as evidence of MRD [13, 14].

### Statistics

Data were analyzed with the SPSS 13 statistical package (Chicago, IL, USA). Overall survival (OS) and relapse-free survival (RFS) were calculated using the Kaplan–Meier (KM) estimates. OS was defined as the time from HSCT to death from any cause, and RFS was defined as the time from HSCT to relapse. The log-rank test was used for the comparison of KM estimates between different groups of patients with a significance level of 0.05. Proportional hazards were calculated with the semi-parametric Cox regression model. Relapse was defined as recurrence of BM blasts >5%, reappearance of blasts in the blood, or development of extramedullary disease infiltrates at any site, Non-relapse mortality (NRM) defined as death without evidence of disease recurrence post-HSCT.

## SUPPLEMENTARY FIGURE AND TABLE


