# Supplementary material for: A 5-day cytoreductive chemotherapy followed by haplo-identical hsct (FA5-BUCY) as a tumor-ablative regimen improved the survival of patients with advanced hematological malignancies
Source: Oncotarget. 2016 Oct 1;7(48):78773–86. doi: 10.18632/oncotarget.12383 (PMC5346676; doi:10.18632/oncotarget.12383)
Supplement: Supplementary file 1 [file oncotarget-07-78773-s001.pdf]

A 5-day cytoreductive chemotherapy followed by haplo-identical hsct (FA5-BUCY) as a tumor-ablative regimen improved the survival of patients with advanced hematological malignancies

SUPPLEMENTARY FIGURE AND TABLE

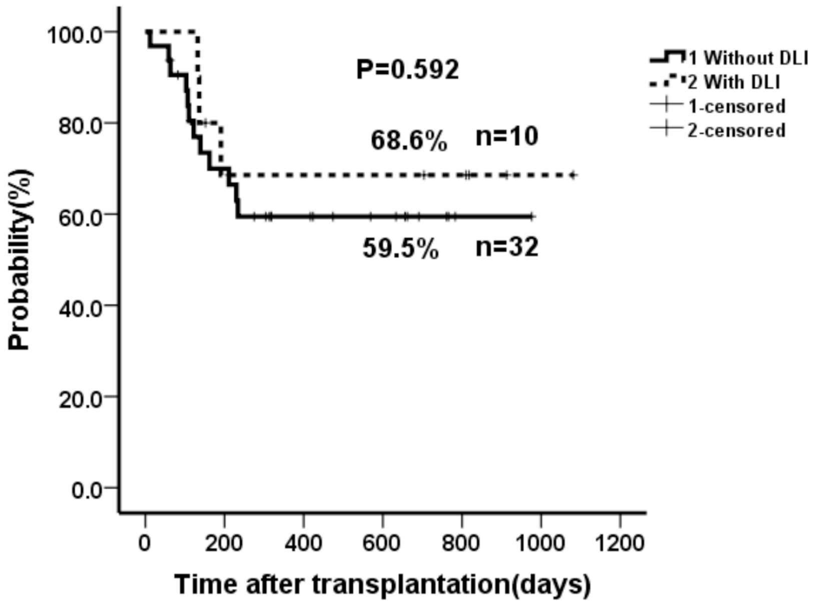

Supplementary Figure S1: The impact of pDLI on RFS for patients not in remission. The relapse free survival in the subgroup with pDLI (n=10) was not significantly better than that in patients without pDLI (n=32) when taking into account all 42 patients not in remission (2-year RFS of 68.6% versus 59.5 %, p=0.592).

Supplementary Table S1: Incidence of acute and chronic GVHD

	No. of patients (%)	
Acute GVHD	I/II	III/IV
Skin	7 (11.1%)	0
Liver	6 (9.5%)	2 (3.2%)
Gut	2 (3.2%)	2 (3.2%)
Chronic GVHD		
Limited	6 (9.5%)	0
Extensive	0	0

Acute GVHD was graded according to the Glucksberg criteria. Chronic GVHD was graded according to the revised Seattle classification.
